# L-Arginine as an Adjuvant Chemosensitizer: Enhancement of Intestinal Permeability and Cytotoxic Activity of Doxorubicin

**DOI:** 10.3390/ph19040546

**Published:** 2026-03-28

**Authors:** Ghada Saad, Rana M. Alquwayi, Hanin B. Alanazi, Farah B. Aldahmashi, Aryam M. Alahmary, Shouq K. Almutairi, Fatima R. Alshammari, Ghadah T. Alshammari, Afnan J. Alrashidi, Norah K. Aldousari, Haifa F. Alsubiei, Lama H. Alanazi, Meaad H. Aldossary, Amal A. Sultan

**Affiliations:** 1 Department of Pharmaceutical Technology, College of Pharmacy, Horus University, New Damietta 34517, Egypt; ghelmoursy@horus.edu.eg; 2 Department of Pharmacy Practice, College of Pharmacy, University of Hafr Al-Batin, Hafr Al-Batin 39911, Saudi Arabia; ralqwayi@gmail.com (R.M.A.); hanenbader2020@gmail.com (H.B.A.); farahaldahmashi1@gmail.com (F.B.A.); alahmaryaryam1@gmail.com (A.M.A.); shoogka49@gmail.com (S.K.A.); fatimaalshammari001@gmail.com (F.R.A.); s2231006211@uhb.edu.sa (G.T.A.); jzamafnan@gmail.com (A.J.A.); norahaldousari4@gmail.com (N.K.A.); hiha8772@gmail.com (H.F.A.); alannziii42@gmail.com (L.H.A.); s2231001433@uhb.edu.sa (M.H.A.); 3 Department of Pharmaceutics, College of Pharmacy, University of Hafr Al-Batin, Hafr Al-Batin 39911, Saudi Arabia; 4 Department of Pharmaceutical Technology, Faculty of Pharmacy, Tanta University, Tanta 31527, Egypt

**Keywords:** doxorubicin, L-arginine, permeability, cytotoxicity, breast cancer

## Abstract

**Background/Objectives**: Doxorubicin is an anthracycline chemotherapeutic agent widely used in the treatment of breast cancer. However, its clinical utility is limited by the drug’s resistance development, low oral bioavailability, and dose-dependent side effects. The semi-essential amino acid, L-arginine, has gained attention as a potential adjuvant that could improve the drug distribution and cytotoxic effectiveness of chemotherapeutics. This study aimed to explore the multifunctional effect of L-arginine on the intestinal absorption and anti-breast cancer activity of doxorubicin. **Methods**: The rabbit in situ intestinal perfusion technique was employed to investigate the membrane transport parameters of doxorubicin both in the absence and presence of L-arginine. Furthermore, the effect of L-arginine on the cytotoxic activity of doxorubicin against breast cancer cells (MCF-7) was assessed using the MTT assay. **Results**: Co-perfusion of L-arginine with doxorubicin enhanced the fraction of doxorubicin absorbed, with a recorded 4.3-fold enhancement in the jejuno-ileum and a 1.5-fold enhancement in the colon segment. In MCF-7 cells, co-treatment with L-arginine resulted in a significant potentiation of doxorubicin cytotoxicity. At L-arginine concentrations of 10 μM and 50 μM, the recorded IC_50_ decreased from 41.3 μM to 8.2 μM and to 22.1 μM, respectively. The superior efficacy of 10 μM L-arginine compared to 50 μM reflected a biphasic concentration-dependent response. **Conclusions**: L-arginine modulated two critical aspects of doxorubicin efficacy, intestinal absorption and cytotoxic activity. The biphasic response emphasizes the importance of L-arginine dose optimization. These findings support the potential of L-arginine as a safe adjuvant for developing oral doxorubicin formulations. This approach can reduce the dose-related toxicity of doxorubicin and improve therapeutic outcomes.

## 1. Introduction

Breast cancer is the most common cancer among women worldwide. Furthermore, it is estimated that about 30% of patients eventually develop an incurable metastatic disease, with an average life expectancy of just over three years [1]. Systemic chemotherapy is currently regarded as the most efficacious treatment for metastatic breast cancer [2]. Despite considerable progress in the development of anticancer agents, conventional pharmaceutical combinations continue to be the primary treatment for breast cancer [3]. Doxorubicin (DOX)-based regimens are among the most compelling strategies to enhance clinical outcomes in the treatment of metastatic breast malignancies [4,5]. DOX is a highly potent anticancer agent belonging to the group of anthracycline glycoside antibiotics. It exerts its cytotoxic activity through multiple mechanisms, including DNA intercalation, topoisomerase II enzyme inhibition, and induction of cellular oxidative damage [6,7]. However, DOX-based chemotherapy exhibits considerable dose-dependent side effects on healthy tissues associated with inadequate tumor selectivity [8,9]. Cardiotoxicity, hepatotoxicity, nephrotoxicity, and myelosuppression are among the major dose-dependent adverse effects that are closely linked to its use [9,10]. Additionally, DOX is primarily available in injectable dosage forms, which necessitate administration under medical supervision. This negatively impacts patients’ quality of life [11]. Therefore, the development of an oral delivery system of doxorubicin may provide advantages to its therapeutic regimens [11,12]. This approach may improve patient compliance, cost effectiveness and avoid infusion-related problems [13,14]. Moreover, oral delivery can result in reduced toxicity and enhanced cytotoxic drug efficacy due to extended systemic exposure and less variation in plasma concentration [15,16]. One of the primary obstacles to effective oral formulation development is the reduced oral bioavailability displayed by most antineoplastic drugs [16,17].

According to Kim et al. (2013), doxorubicin is classified as a BCS class III drug due to its high solubility and low membrane permeability [18]. The poor oral absorption of doxorubicin was previously thought to be caused by cytochrome P450 (CYP3A4) metabolism and P-gp efflux [11]. Furthermore, it has been found that cellular internalization of doxorubicin is influenced by multidrug resistance protein 1 (MRP1) and breast cancer resistance protein (BCRP) efflux transporters [19,20,21]. Efflux inhibitors can increase the intestinal absorption of substrates for efflux transporters [11]. The oral bioavailability of drugs can be increased by carefully selecting the formulation excipients [22]. A fundamental step in the rational design of advanced drug delivery systems is the introduction of new absorption enhancers as pharmaceutical excipients [23]. Surfactants, bile salts, chelating agents, and fatty acids have been adopted as absorption enhancers. However, the intestinal mucosal barrier is frequently harmed and irritated by these agents. For usage in clinical practice, it is necessary to design absorption enhancers that are both effective and less harmful [24].

L-arginine is a semi-essential amino acid playing a vital role in various physiological processes. L-arginine, with its positively charged guanidinium group, is the backbone of many cell-penetrating peptides (CPPs) [25]. It serves as the primary physiological precursor of nitric oxide (NO). It is now clear that NO has a variety of functions in the gastrointestinal tract’s physiology and pathology [26]. Beyond its proposed role to enhance oral absorption, L-arginine has been reported to make cancer cells more vulnerable to standard chemotherapy [27]. This would shift the scales in favor of cancer treatment while sparing the healthy cells [28]. As the physiological precursor of NO synthesis, L-arginine can remodel the tumor microenvironment (TME) and make DOX work better. Moreover, L-arginine feeds the immune soldiers in the TME, such as T-cells, NK cells, and other immune cells, boosting their anti-tumor effect [29]. However, the intricate relationships between various NO levels and many aspects of tumor formation and progression have produced seemingly contradictory results [30].

Accordingly, this study aims to investigate the potential of L-arginine as a dual-acting adjuvant to enhance both the intestinal absorption and cytotoxic activity of doxorubicin. An in situ intestinal perfusion technique was utilized to explore the effect of L-arginine on the intestinal absorption of DOX. This employed albino rabbits as test animals. Additionally, the effects of combinatorial treatment with L-arginine to enhance the chemosensitivity of doxorubicin in MCF-7 breast cancer cells were investigated.

## 2. Results

### 2.1. Chromatography

For the analysis of doxorubicin at pH values of 6.6 and 7.4, a high-performance liquid chromatographic analytical method was appropriately utilized. Following retention periods of 3.1 min at pH 6.6 and 2.9 min at pH 7.4, the drug was eluted. In the concentration range of 3 to 25 µg/mL, the created calibration graphs were linear. The regression equations derived were Y = 430.98X + 345.82 (R^2^ = 0.997) at pH 6.6, and Y = 423.06 X + 313.78 (R^2^ = 0.999) at pH 7.4. These were used to quantify doxorubicin at the corresponding pH levels. The percentage of the drug retrieved in relation to the nominal concentration verified the assay’s accuracy. At pH 6.6 and pH 7.4, the calculated percentage recovery values varied from 94.2% to 108.2% and from 97.3% to 104.14%, respectively. The calculated relative standard deviation (RSD) values ranged from 0.2% to 2% at pH 6.6 and from 0.4% to 3.1% at pH 7.4. These values demonstrated the accuracy of the formulated assay procedure. At pH 6.6 and pH 7.4, the computed LOD values were 0.4 μg/mL and 0.6 μg/mL, respectively, and the calculated LOQ values were 1.7 μg/mL and 1.8 μg/mL, respectively.

### 2.2. In Situ Intestinal Absorption Studies

The in situ intestinal perfusion technique in rabbits was employed to simulate the actual absorption of doxorubicin from its aqueous solution within the body, while maintaining the physiological blood flow and innervation of the intestine [11]. This technique allows for a highly accurate assessment of the drug’s absorption, while eliminating the potential influence of food intake and gastric emptying rate, which could otherwise alter absorption outcomes [31]. The use of rabbits as an experimental model further enhances the reliability of this technique, due to the considerable anatomical and functional similarities between the gastrointestinal systems of rabbits and humans, thereby improving the translational relevance of the absorption data [32].

#### 2.2.1. Drug Stability Assessment During Perfusion Experiments

Doxorubicin stability during perfusion studies was assessed through comparison of the drug concentrations in the perfusion solutions before and after each experiment. The drug recovery was calculated as the ratio of final to initial concentration expressed as a percentage. Regarding pure DOX perfusion solutions, the calculated % recovery values were 101.5 ± 5.98 at pH 6.6 and 100.8 ± 4.4 at pH 7.4. For the DOX/L-arginine combination solutions, the % recovery was 97.9 ± 7.3 at pH 6.6, and 99.0 ± 2.97 at pH 7.4. The computed drug recovery data were considered indicative of acceptable doxorubicin stability in all the prepared solutions throughout the perfusion experiment. Accordingly, the recorded reduction in DOX after intestinal perfusion can be attributed to drug absorption rather than drug degradation.

#### 2.2.2. Regional-Specific Intestinal Absorption of Doxorubicin

**Figure 1:**
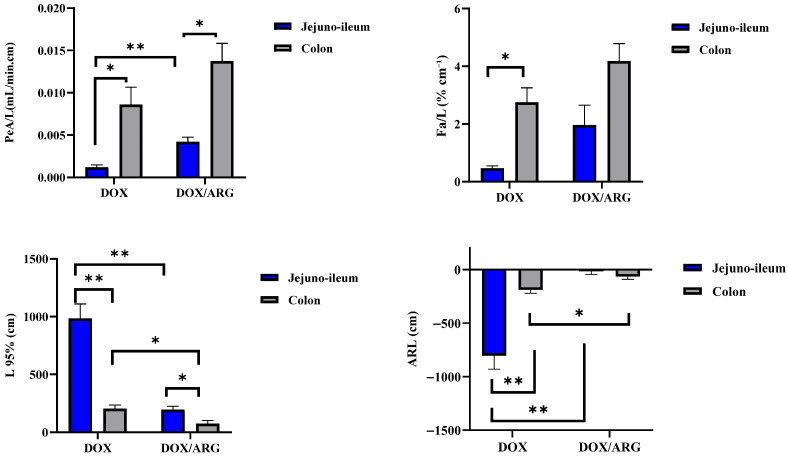
Comparative presentation of in situ membrane transport parameters. All values are expressed as the mean ± SD (n = 3). The significance levels were indicated as * *p* < 0.05 and ** *p* < 0.01.

Intestinal absorption of doxorubicin was assessed at two anatomical regions: the jejuno-ileum and colon segments. The calculated membrane transport parameters are listed in Table 1. A comparative presentation of the recorded data is illustrated in Figure 1.

The recorded absorption parameters for the control doxorubicin solution (DOX) demonstrated incomplete drug absorption. This was evidenced by negative ARL values in both regions. Moreover, doxorubicin absorptive clearance normalized to unit intestinal length (PeA/L) at steady state was significantly influenced by the intestinal site, with markedly greater absorption observed in the colon compared with the jejuno-ileum (*p* < 0.05, Table 1; Figure 1). In addition, the estimated L95% value was significantly lower in the colon segment compared to the jejuno-ileal segment (*p* < 0.01). The recorded mean fraction absorbed per unit intestinal length (%Fa/cm) was significantly higher in the colon segment compared to the jejuno-ileum (*p* < 0.05, Table 1; Figure 1).

**Figure 2:**
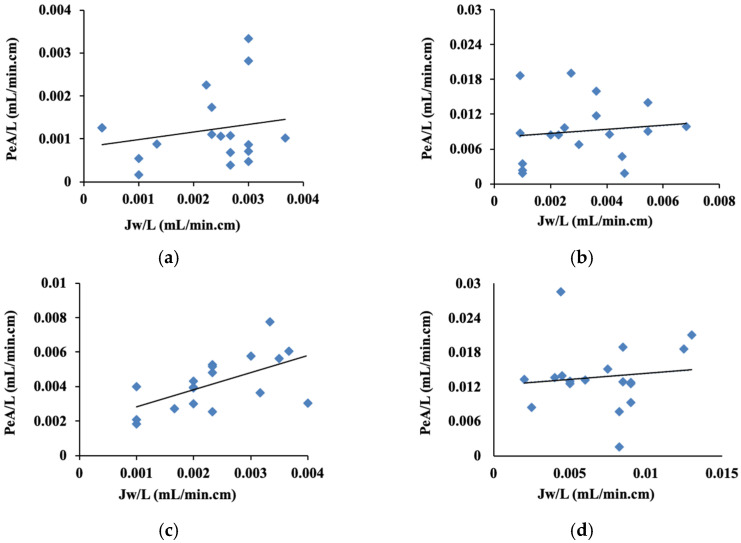
Doxorubicin absorptive clearance versus water flux from the jejuno-ileum (**a**) and colon (**b**) (DOX), and after co-perfusion with L-arginine from the jejuno-ileum (**c**) and colon (**d**) segments (DOX/ARG).

In order to study the drug’s absorptive routes, the impact of water flux on doxorubicin membrane transport was tracked. This was accomplished by plotting the drug absorptive clearance at steady state against net water flux, both normalized to segment length (Figure 2). The proportional contributions of transcellular and paracellular routes to doxorubicin absorption were determined using linear regression analysis of the created plots. The intercept, which supplied the transcellular absorption at zero water flux, was used to estimate the transcellular absorption contribution. The paracellular pathway’s contribution is indicated by the variation between the intercept and the total absorptive clearance. Table 2 displays these estimated contributions. The computations showed that the transcellular and paracellular routes contributions were 66.6% and 33.4%, respectively, in the jejuno-ileum (Table 2). However, doxorubicin absorption in the colon segment was significantly dependent on the diffusive transcellular pathway (88.2%, Table 2).

#### 2.2.3. Effect of L-Arginine on Intestinal Absorption of Doxorubicin

Doxorubicin intestinal absorption was investigated in the presence of L-arginine in jejuno-ileum and colon segments. Doxorubicin absorption from both investigated segments was significantly increased by co-perfusion with L-arginine (DOX/ARG), with the enhancement being more notable in the jejuno-ileum (Table 1). This showed up as a considerably higher PeA/L and lower length required for 95% drug absorption (L95%) (*p* < 0.01, Figure 1). The recorded ARL value approached zero, indicating nearly complete DOX absorption after co-perfusion with L-arginine in the jejuno-ileum (Table 1, Figure 1). The colon segment revealed a significant reduction in the recorded L95% (*p* < 0.05). Regarding the absorptive pathways of doxorubicin from the jejuno-ileum in the presence of L-arginine, the transcellular and paracellular pathways contributed 42.9% and 57.1%, respectively (Table 2). The transcellular diffusive pathway predominated in the colon segment (89.6%, Table 2).

### 2.3. Cytotoxicity Studies Using MTT Assay

**Figure 3:**
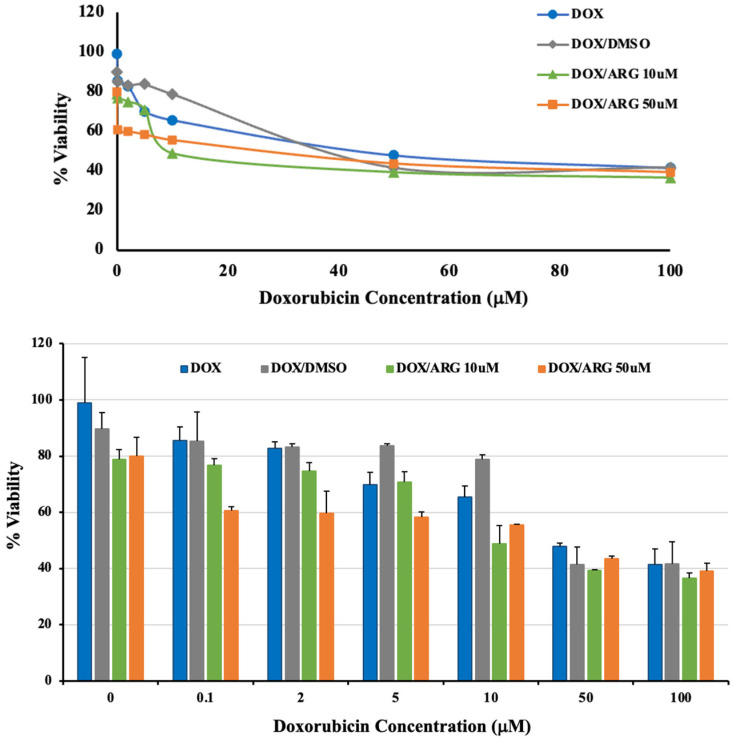
The effect of L-arginine on cytotoxic activity of doxorubicin on MCF-7 cells. Error bars represent the SD values (n = 3).

Using an MTT test, the cytotoxic efficacy of free doxorubicin was assessed on breast cancer cells (MCF-7) both with and without L-arginine at two concentrations (10 μM and 50 μM). The percentage vitality of the treated cells in comparison to untreated control cells was defined as cytotoxicity. The evaluated percentages of cell viability in the presence of doxorubicin at concentrations of 0, 0.1, 1, 2, 5, 10, 50, and 100 μM are shown in Figure 3. For free doxorubicin (DOX), the calculated IC_50_ value was 41.3 μM. The presence of DMSO in the positive control resulted in a non-significant reduction in the recorded IC_50_ of doxorubicin (39.7 μM). However, co-treatment of MCF-7 cells with L-arginine significantly enhanced doxorubicin’s cytotoxic effects, as revealed from the marked reductions in IC_50_ values. At 10 μM L-arginine (DOX/ARG 10μM), the computed doxorubicin IC_50_ value dramatically dropped to 8.2 μM (*p* < 0.01), representing a 4.8-fold enhancement of cytotoxic potential compared to doxorubicin alone. At 50 μM L-arginine (DOX/ARG 50μM), a 1.8-fold enhancement in doxorubicin cytotoxicity was recorded, with the computed IC_50_ value being 22.1 μM (*p* < 0.05).

## 3. Discussion

Breast cancer is one of the leading causes of mortality for women worldwide. This is attributed to its aggressive nature, early metastases, and resistance to conventional chemotherapy. One of the most effective therapies for breast cancer is doxorubicin (DOX), a potent anticancer medication [33]. Several approaches have been used to reduce the dose-dependent side effects of DOX. These include the use of substances with antioxidant and/or antiapoptotic effects, the development of efficient delivery systems, the investigation of prodrugs, and the synthesis of DOX analogs [34,35].

L-arginine has emerged as an adjuvant capable of enhancing the permeation of drugs across biological barriers. Additionally, the role of L-arginine in enhancing the cytotoxic activity of chemotherapeutics has been reported for various cancer models [36,37,38]. The present study investigates the effect of L-arginine on the intestinal absorption and cytotoxic potential of doxorubicin against breast cancer cells.

The in situ intestinal perfusion technique in rabbits was employed to explore the intestinal absorption of doxorubicin from its aqueous solution, both in the absence and presence of L-arginine. In situ intestinal perfusion studies revealed limited membrane permeability of DOX from its aqueous solution in both jejuno-ileum and colon segments. These findings are consistent with previous reports classifying doxorubicin as a Biopharmaceutics Classification System (BCS) class III compound characterized by limited biological membrane penetration [11]. Moreover, the recorded membrane transport parameters reflected the site-dependent absorptive clearance of DOX. The recorded parameters revealed superior DOX absorption from the colon segment. This site-dependent absorption profile is consistent with the established role of DOX as a substrate for P-glycoprotein (P-gp), together with the higher expression of P-gp efflux transporters in the jejuno-ileum segment [11]. Additionally, other investigators have reported doxorubicin as an MRP1 and BCRP substrate [19,20,21,22]. It was observed that doxorubicin absorption in the colon was superior to that in jejuno-ileum, likely due to the regional variation in transporter expression within the small intestine contributing to differences in absorption. Furthermore, the effect of water flux on the absorption of doxorubicin was monitored by analyzing the relationship between absorptive clearance and water flux. The results demonstrated that DOX absorption was predominantly via the diffusive transcellular pathway in both investigated intestinal segments. Co-perfusion of DOX with L-arginine at a 1:1 molar ratio resulted in a significant enhancement of the intestinal permeability of DOX in both the jejuno-ileum and colon segments. This may be attributed to the unique structure of L-arginine with its guanidinium group. Previous studies highlighted the potential of cell-penetrating peptides and other guanidinium-rich compounds such as L-arginine to enhance the endocytosis of a variety of drugs and biomarkers. It has been proposed that the transport toward the cell interior is facilitated by a transient membrane channel created when deprotonated fatty acids on the cell membrane interact with guanidinium groups [39,40]. Another possible explanation may be related to the role of L-arginine as the biological precursor of nitric oxide (NO) [41]. NO is involved in the modulation of smooth muscle tone in the gastrointestinal tract (GIT), which includes the control of antral motor activity, gastric emptying, and intestinal peristalsis. It also controls the synthesis of alkali, the secretion of gastric and acid mucus, and the preservation of mucosal blood flow [42]. Its vasodilating effect permits the maintenance of blood flow that may facilitate passive diffusion of DOX across the intestinal epithelium. According to earlier studies, NO generated locally by activated enterocytes may contribute to barrier dysfunction. This can directly improve the permeability of the enterocyte monolayer and the ileal mucosal membrane [43].

The current study demonstrated that L-arginine potentiates the cytotoxicity of DOX against MCF-7 breast cancer cells. Treatment of the test cells with DMSO vehicle revealed no significant alteration in cytotoxicity compared to DOX in normal saline. This highlighted that the observed enhancements are attributable to L-arginine rather than the added vehicle. Combination of DOX with 10 μM L-arginine resulted in a significant intensification of DOX cytotoxicity, with a 4.8-fold enhancement being recorded. However, increasing L-arginine concentration to 50 μM demonstrated a 1.8-fold enhancement of DOX cytotoxicity, reflecting a biphasic response. Previous studies reported that L-arginine alone did not significantly affect the viability of MCF-7 cells at concentrations up to 200 μg/mL (≈1150 μM) [44]. Moreover, other studies have demonstrated that arginine is essential for the survival and proliferation of MCF-7 cells [45]. This reflects that the superior cytotoxicity of DOX in the presence of L-arginine can be attributed to the chemosensitizing effect of L-arginine rather than to a direct toxic effect of the amino acid itself. Previous investigators reported that the guanidinium group of L-arginine might be a key component of its potential mechanism of action. Endothelial production of NO from terminal guanidino nitrogen atoms of L-arginine enhances vascular permeability, lowers leukocyte–endothelial interactions, and increases and/or maintains tumor blood flow. Accordingly, increasing tumor perfusion may improve drug distribution and accumulation in the tumor tissue, possibly enhancing therapeutic effects without causing systemic toxicity. However, this requires further investigations, including direct measurements of NO production and intracellular DOX accumulation. Furthermore, previous findings suggested that arginine might cause tight junction proteins to internalize into the subcellular space, which in turn increased the Caco-2 cell monolayer’s permeability through a paracellular pathway [46].

On the other hand, higher L-arginine concentrations may trigger pro-survival metabolic and signaling pathways that offset the cytotoxic effects of doxorubicin. In addition to serving as a substrate for the synthesis of NO, L-arginine also directly activates nutrient-sensing pathways like mTORC1. Recent studies have reported that mTORC1 activation in response to amino acids, particularly arginine, subsequently improves cell viability [47]. Increased arginine availability can stimulate mTOR signaling, which is linked to decreased apoptotic responses, increased protein synthesis, and cellular growth and survival signaling in cancer cells [48,49]. While low concentrations make cancer cells more sensitive to chemotherapy, higher concentrations may cause cellular metabolism and signaling to change in favor of survival and resistance [50]. This dual effect highlights the importance of metabolic context in drug combination studies as well as arginine’s intricate role as a nutrient and signaling metabolite in tumor biology. However, this requires further investigations. Moreover, formal combination index analysis should be performed in future studies employing wider concentration ranges to identify the optimal synergistic ratio of L-arginine to doxorubicin.

## 4. Materials and Methods

### 4.1. Materials

Hospira (HSP) Pharmaceutical and Medical Company, Cairo, Egypt, provided doxorubicin HCl as a gift, while Sigma Aldrich Chemical Co., St. Louis, MO, USA, supplied L-arginine and acetonitrile (HPLC-grade). El-Nasr Pharmaceutical Chemicals Co., Cairo, Egypt, kindly provided the potassium di-hydrogen phosphate, di-sodium hydrogen phosphate, sodium chloride, potassium chloride, ethanol, sodium lauryl sulphate, hydrochloric acid, and ortho-phosphoric acid.

### 4.2. Chromatography

Doxorubicin was quantitatively determined using high-performance liquid chromatographic analysis (HPLC). This employed a Shimadzu HPLC system (KNAUER AZURA P6.1L, Berlin, Germany) equipped with a manual sampling system (KNAUER, D-14163, Berlin, Germany) and a UV–Vis spectrophotometric detector (AZURA UVD 2.1L detector, KNAUER, Berlin, Germany). A reversed-phase C-18 column of 150 mm in length, 4.6 mm in internal diameter, and an average particle size of 5 μm was used for the separation process (Knauer Eurospher II 100–5, KNAUER, Berlin, Germany). The mobile phase comprised a mixture of acetonitrile and an aqueous buffer at a 1:1 volume ratio. The buffer consisted of 0.05 M di-sodium hydrogen phosphate and 0.1% *w*/*v* sodium lauryl sulphate solution, adjusted to pH 3.7 with o-phosphoric acid. The mobile phase was pumped at a flow rate of 0.6 mL/min, and the effluent was detected at a wavelength of 233 nm. The whole system control, including data analysis, was performed by Knauer Clarity Chrome software (KNAUER, Berlin, Germany; https://www.knauer.net, accessed on 27 February 2026). The method was validated according to the ICH guidelines. The validation metrics were sensitivity (lower limit of detection and lower limit of quantification), linearity, accuracy, precision, and selectivity.

### 4.3. Preparation of Perfusion Solutions

Doxorubicin was incorporated in the perfusion solutions at a concentration of 20 μg/mL. Perfusion solutions were prepared both with and without L-arginine. Phosphate-buffered saline (PBS) was chosen as a vehicle for perfusion solutions based on its isotonic osmolarity, buffer properties and stabilized pH during perfusion experiments. It had a composition of 137 mM sodium chloride, 2.7 mM potassium chloride, 10 mM disodium hydrogen phosphate, and 1.8 mM potassium dihydrogen phosphate. For L-arginine perfusion solutions, L- arginine was added at a 1:1 molar ratio with doxorubicin, corresponding to a concentration of 6.4 μg/mL. The pH of the prepared solutions was maintained at 7.4 for jejunoileal perfusion and was adjusted to 6.6 for colonic perfusion. All the prepared solutions were warmed to 37 ± 0.5 °C prior to in situ perfusion. This was performed to mimic the physiological pH and temperature of the perfused intestinal segments [11].

### 4.4. In Situ Intestinal Absorption Studies

**Figure 4:**
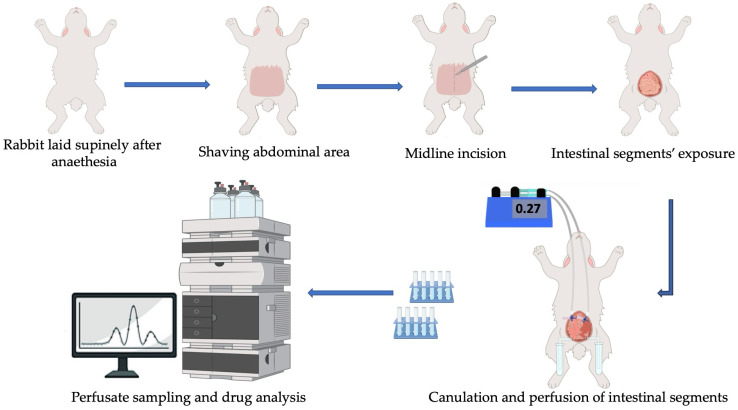
Schematic illustration of surgical procedures and the experimental setup of the in situ intestinal perfusion technique.

Doxorubicin’s intestinal permeability was tracked using the in situ intestinal perfusion technique. Six albino rabbits with an average weight of 2.25 ± 0.25 kg were used in this study. The animal study protocol was approved by the Central Research Ethics Committee, Scientific Research Unit, Horus University, Egypt (Approval code: PH-2026-016). This procedure adhered to the National Institutes of Health’s guidelines for the use and care of laboratory animals. The surgical procedures of the in situ intestinal perfusion technique are illustrated in Figure 4.

The rabbits were randomly allocated into two experimental groups (n = 3 per group). The first group served as the control group to investigate the intestinal absorption of pure doxorubicin from its aqueous solution (20 μg/mL). The other group served as the test group and was perfused with doxorubicin and L-arginine at a 1:1 molar ratio. The intestinal segments of the colon and jejuno-ileum were employed to monitor the absorption of doxorubicin. The surgical techniques used to expose the targeted intestinal segments have been established and described in prior studies [51,52]. Following an overnight fast, the tested rabbits were anesthetized by two consecutive intramuscular injections of ketamine HCl (45 mg/kg each) administered at 15 min intervals. If necessary, a further ketamine HCl injection of 25 mg/kg was administered. Before each dosage of anesthesia, a muscle relaxant injection of chlorpromazine HCl was administered [53]. The tested rabbit was positioned supinely on a warmed pad, and the abdominal area was shaved. A midline longitudinal abdominal incision was then made to expose the intestinal segments of interest. A 30 cm jejuno-ileal segment was cautiously isolated and cannulated at the proximal end using a three-way stopcock cannula. The distal end was cannulated with an L-shaped glass cannula to allow outflow of the perfusate. Warm normal saline was used to carefully clean the exposed segment’s lumen. The 10 cm colon segment was similarly prepared and exposed. To guarantee a constant flow of the perfusion solution, the accessible test segments were arranged in a consistent S- to multi-S-pattern. To preserve physiological conditions and tissue viability during perfusion experiments, the exposed segments were covered with a gauze pad that was frequently soaked with warm saline. Using a perfusion pump (Harvard-22 Apparatus, Millis, MA, USA), the tested solutions were perfused at a flow rate of 0.27 mL/min. The effluent was collected in drainage tubes at 10 min intervals over a period of two hours. In accordance with previously established protocols, steady-state conditions were attained during the second hour of intestinal perfusion [54]. The volume of each sample was accurately measured using a graduated syringe. The samples were then centrifuged to remove any mucus debris, and the resulting supernatant was injected (30 μL) into HPLC for drug detection. Lastly, the tested animal was euthanized, the intestinal segments that had been cannulated were removed, and their actual length was precisely measured. All assays and experimental procedures were shielded from direct light exposure by covering them with black plastic film to protect the drug from photodegradation.

### 4.5. Data Analysis

#### 4.5.1. Absorptive Clearance

The concentration of the drug in the perfusate was normalized to account for net water flux. The experimentally measured sample volumes were compared to the theoretical volumes (based on the calibrated pump flow rate and collection time) to estimate the net water flux (Jw). The fraction of drug remaining in the effluent perfusate was determined by calculating the ratio of the normalized outflow concentration (C_(out)_) to the inflow perfusion concentration (C_(in)_). The remaining fractions of the drug in the steady-state samples expressed as (C_(out)_/C_(in)_)ss were computed and averaged. Equations (1) and (2) were utilized to determine the drug’s absorptive clearance (PeA) in mL/min from the outflowing samples during the second hour of the perfusion study:

(C_(out)_/C_(in)_)ss = exp^−(PeA/Q)^ (1)

PeA = −Q.ln(C_(out)_/C_(in)_)ss (2)

where Q is the average flow rate of the perfusion solution (mL/min), A is the effective surface area (cm^2^), and Pe is the apparent permeability coefficient (cm/min).

The fraction absorbed of the drug (Fa) can be estimated indirectly from the fraction remaining at steady state using the following equation:

Fa = 1 − {C_(out)_/C_(in)_}ss = 1 − exp^−(PeA/Q)^ (3)

Anatomical reserve length (ARL) is the remaining intestinal length after complete drug absorption. ARL was used to express the extent of intestinal drug absorption, with positive values being taken as an indicator of the segment’s complete drug absorption. ARL is given by the following equation:

ARL = L* − l*(4)

where L* denotes the anatomical maximum length of the tested intestinal segment in cm, and l* represents the experimentally assessed intestinal length required for complete drug absorption in cm.

Taking into consideration the logarithmic nature of the mentioned equations, the concentration will approach zero only and will not deplete completely until it reaches zero at the intestinal length (l*). Accordingly, a 5% residual proportion of the drug that was perfused was formerly thought to indicate roughly full drug absorption. The following equation was used to estimate l*:

0.05 = exp^−(PeA.l*/Q)^ (5)

where PeA is the same as in equation (1) but normalized to length, and l* is the length required for 95% absorption (L95%) of the drug.

#### 4.5.2. Effect of Water Flux on Intestinal Drug Absorption

The total intestinal absorption of drugs is influenced by both diffusive and convective drug transport mechanisms. Consequently, Equation (6) can be used to express the rate of drug absorption from intestinal lumen per unit time (Js).

Js = K_s_(C − C_p_) + Φ_s_JwC (6)

where Js is the absorbed drug per unit time in (mg/min) and [K_S_(C − C_p_)] denotes the diffusive process, while [Φ_S_JwC] presents the convective process. K_S_ and Φ_S_ are the diffusive permeability coefficient and the drug sieving coefficient, respectively. Jw is the net water flux, which is an influx–outflux process. C is the drug concentration in the lumen and C_P_ is the drug concentration in the plasma. Considering that sink conditions were preserved and steady-state conditions were attained during the second hour of perfusion, the prior equation can be modified as follows:

Jss = K_s_Css + Φ_s_JwCss (7)

where Css is the residual intestinal drug concentration at steady state (mg/mL) and Jss is the drug flow at steady state (mg/min). Equation (8) is obtained by rearranging the previous equation, where Jss/Css represents the total drug absorptive clearance in (mL/min) independent of the drug absorption mechanism and can be measured empirically as (PeA) (see Equation (2) above):

Jss/Css = K_s_ + Φ_s_Jw (8)

A linear plot was produced by graphic regression analysis of the dependent variable (PeA) against the net water flux (Jw). This line’s slope can be used to calculate the absorptive clearance’s convective part, and its intercept with the (Y axis) can be used to estimate the diffusive part.

### 4.6. Drug Stability Assessment During Perfusion Experiments

Doxorubicin’s stability under experimental conditions can be assessed through a comparison of the drug’s concentrations in the perfusion solutions before and after each experiment. Samples were collected directly from the syringe of the perfusion pump before starting perfusion and immediately after the collection of the last perfusate sample. Doxorubicin initial (*C*_initial_) and final (*C*_final_) concentrations were quantified using HPLC. The % recovery was calculated using the following equation:

$$\mathrm{Recovery}\;(\%)=\left(\frac{C_{\mathrm{final}}}{C_{\mathrm{initial}}}\right)\times 100$$

A recovery of ≥90% of the initial concentration was considered indicative of acceptable doxorubicin stability throughout the perfusion experiment [55].

### 4.7. Cytotoxicity Study of Doxorubicin by MTT Assay

#### 4.7.1. Cell Culture

The human breast cancer cell line MCF-7 was cultured in RPMI-1640 (Roswell Park Memorial Institute, Buffalo, NY, USA) medium supplemented with 10% (*v*/*v*) heat-inactivated fetal bovine serum (FBS) and 1% penicillin–streptomycin solution (100 U/mL penicillin and 100 μg/mL streptomycin). Cells were maintained in a humidified incubator at 37 °C under an atmosphere of 95% air and 5% CO_2_, with the culture medium replaced every other day.

The cultivated cells were sub-cultured using 0.25% trypsin with 0.02% EDTA in PBS for three minutes at 37 °C once they were 95% confluent. Using an 8-channel micropipette, the separated cells were resuspended at a concentration of 4 × 10^4^ cells/cm^2^ and then placed in a 96-well plate (200 μL/well). Blank control wells without cultured cells were used as background controls for absorbance measurements.

#### 4.7.2. Cell Viability Analysis Using MTT Assay

Cell viability was assessed using MTT assay to investigate the effect of L-arginine at two concentrations (10 μM and 50 μM) on the cytotoxic activity of doxorubicin. The MTT assay is a reliable, quantitative, and sensitive colorimetric method that measures cellular metabolic activity and determines cell viability. Furthermore, the assay is based on the ability of mitochondrial lactate dehydrogenase enzymes (LDH) in viable cells to reduce the water-soluble substrate 3-(4,5-dimethylthiazol-2-yl)-2,5-diphenyl tetrazolium bromide (MTT) into dark blue water-insoluble formazan crystals.

The cytotoxic impact of doxorubicin was examined at concentrations of 0, 0.1, 1, 2, 5, 10, 50, and 100 μM in two different vehicles: saline and DMSO. For the DMSO group, the final DMSO concentration in all treatment wells was kept below 0.5% (*v*/*v*) to avoid solvent-induced cytotoxicity. Doxorubicin cytotoxicity was investigated both in the presence and absence of L-arginine at two concentrations: 10 μM and 50 μM. The cells were treated with the tested concentration and incubated for 48 h at 37 °C. Following the removal of the dead cells using PBS at a pH of 7.2 ± 0.2 (PBS-0.05% Tween), the treated cells were examined under a microscope to look for any morphological changes.

To assess cell survival, a 5 mg/mL (pH 7.2 ± 0.2) MTT solution was made in PBS, and 25 μL of this solution was applied to each well. The wells were then incubated for 3–4 h at 37 °C. For 30 min on a plate shaker, a solubilization flush of dimethyl sulfoxide (DMSO, 0.05 mL) was added to fully dissolve the formed intracytoplasmic MTT formazan crystals into a colored solution. Each well’s optical density (OD) value was measured spectrophotometrically at a wavelength typically ranging between 500 and 600 nm using an ELISA microplate reader (Biotek-800 TS, BioTek Instruments, Inc., Winooski, VT, USA) [56,57]. The IC_50_ values of the test samples were calculated using the MasterPlex 2010 software program. Data were obtained from three independent experiments (n = 3) and are presented accordingly [11,58]. The amount of doxorubicin needed to inhibit 50% of cell growth in comparison to the blank control value is known as the computed IC_50_ value. The following formula was used to determine relative viability as a percentage [59,60]:

Cell viability (%) = (OD of treated cells/OD of untreated cells) × 100

### 4.8. Statistical Analysis

All data were presented as average ± standard deviation (n = 3). A one-way analysis of variance (ANOVA) followed by Tukey’s Post Hoc was employed for multiple comparisons. Differences were considered statistically significant at *p* values < 0.05. This utilized GraphPad Prism version 8.0.0 (GraphPad Software, San Diego, CA, USA).

## 5. Conclusions

The typical chemotherapeutic agent doxorubicin (DOX) is a potent anticancer drug. It is widely used in the treatment of numerous cancer types including breast cancer. Its clinical effectiveness is frequently limited by dose-dependent adverse effects, the development of drug resistance, and limited oral bioavailability. This study investigated the potential of L-arginine as an adjuvant chemosensitizer to enhance the intestinal absorption and cytotoxic activity of doxorubicin. Absorption studies employing the in situ rabbit intestinal perfusion technique revealed limited membrane absorption of DOX. Co-perfusion with L-arginine resulted in significant enhancement of the membrane transport parameters of DOX from the jejuno-ileum and colon. Furthermore, the effect of L-arginine on the cytotoxic effect of DOX against breast cancer cells (MCF-7) was assessed using the MTT assay. Treatment of cells with doxorubicin/arginine combinations significantly enhanced cytotoxicity compared to pure DOX. The overall findings establish L-arginine as a promising chemotherapy adjuvant for improving both the oral absorption and cytotoxic efficacy of doxorubicin. However, the findings of the current study are limited to MCF-7 cells and require validation in additional breast cancer cell lines.

## Figures and Tables

**Table 1:** Doxorubicin’s membrane transport parameters in the jejuno-ileum and colon segments.

	Jejuno-Ileum				
	PeA/L (mL/min.cm)	%Fa/L (%cm^−1^)	L95% (cm)	ARL	Jw/L (mL/min.cm)
DOX	0.0012 ± 0.0005	0.46 ± 0.16	984.90 ± 216.7	−804.90 ± 216.7	0.004 ± 0.002
DOX/ARG	0.0042 ± 0.0010	1.96 ± 0.70	195.95 ± 49.8	−15.95 ± 49.8	0.002 ± 0.0002
	**Colon**				
	PeA/L (mL/min.cm)	%Fa/L (%cm^−1^)	L95% (cm)	ARL	Jw/L (mL/min.cm)
DOX	0.0086 ± 0.0036	2.75 ± 0.86	203.74 ± 57.3	−193.74 ± 57.3	0.0031 ± 0.0014
DOX/ARG	0.0137 ± 0.0036	4.18 ± 1.05	74.91 ± 25.3	−64.91 ± 25.3	0.0071 ± 0.0022

L95% is the estimated length needed for 95% doxorubicin absorption, ARL is the anatomical reserved length, JW/L is the water flux per unit intestinal length, PeA/L is the doxorubicin absorptive clearance normalized to the explored segment length, and %Fa/L is the fraction of drug absorbed expressed as percentage per unit length. The values are shown as mean ± SD (n = 3).

**Table 2:** Transcellular and paracellular pathways’ respective contributions to the absorption of doxorubicin from aqueous solution (DOX) and doxorubicin/L-arginine solution (DOX/ARG).

Perfusion Solution	Jejuno-Ileum	
	% Transcellular Absorption	% Paracellular Absorption
DOX	66.6%	33.4%
DOX/ARG	42.9%	57.1%
	**Colon**	
	% Transcellular Absorption	% Paracellular Absorption
DOX	88.2%	11.8%
DOX/ARG	89.6%	10.4%

## Data Availability

The original contributions presented in this study are included in the article. Further inquiries can be directed to the corresponding authors.

## References

[1] Redig A.J., McAllister S.S. (2013). Breast cancer as a systemic disease: A view of metastasis. J. Intern. Med..

[2] Cabeza L., Ortiz R., Arias J.L., Prados J., Ruiz Martínez M.A., Entrena J.M., Luque R., Melguizo C. (2015). Enhanced antitumor activity of doxorubicin in breast cancer through the use of poly(butylcyanoacrylate) nanoparticles. Int. J. Nanomed..

[3] Morris P.G., McArthur H.L., Hudis C.A. (2009). Therapeutic options for metastatic breast cancer. Expert. Opin. Pharmacother..

[4] Sinha B.K., Mimnaugh E.G., Rajagopalan S., Myers C.E. (1989). Adriamycin activation and oxygen free radical formation in human breast tumor cells: Protective role of glutathione peroxidase in adriamycin resistance. Cancer Res..

[5] Pilco-Ferreto N., Calaf G.M. (2016). Influence of doxorubicin on apoptosis and oxidative stress in breast cancer cell lines. Int. J. Oncol..

[6] Minotti G., Menna P., Salvatorelli E., Cairo G., Gianni L. (2004). Anthracyclines: Molecular advances and pharmacologic developments in antitumor activity and cardiotoxicity. Pharmacol. Rev..

[7] Schwarzbach M.H., Eisold S., Burguete T., Willeke F., Klein-Bauernschmitt P., Schlehofer J.R., Herfarth C., Ridder R., Von Knebel Doeberitz M. (2002). Sensitization of sarcoma cells to doxorubicin treatment by concomitant wild-type adeno-associated virus type 2 (AAV-2) infection. Int. J. Oncol..

[8] Carvalho C., Santos R.X., Cardoso S., Correia S., Oliveira P.J., Santos M.S., Moreira P.I. (2009). Doxorubicin: The good, the bad and the ugly effect. Curr. Med. Chem..

[9] Toldo S., Goehe R.W., Lotrionte M., Mezzaroma E., Sumner E.T., Biondi-Zoccai G.G., Seropian I.M., Van Tassell B.W., Loperfido F., Palazzoni G. (2013). Comparative cardiac toxicity of anthracyclines in vitro and in vivo in the mouse. PLoS ONE.

[10] Kciuk M., Gielecińska A., Mujwar S., Kołat D., Kałuzińska-Kołat Ż., Celik I., Kontek R. (2023). Doxorubicin—An agent with multiple mechanisms of anticancer activity. Cells.

[11] Sultan A.A., Saad G.A., El Maghraby G.M. (2023). Permeation enhancers loaded bilosomes for improved intestinal absorption and cytotoxic activity of doxorubicin. Int. J. Pharm..

[12] Le Lay K., Myon E., Hill S., Riou-Franca L., Scott D., Sidhu M., Dunlop D., Launois R. (2007). Comparative cost-minimisation of oral and intravenous chemotherapy for first-line treatment of non-small cell lung cancer in the UK NHS system. Eur. J. Health Econ..

[13] Dong Y., Feng S.S. (2005). Poly (d, l-lactide-co-glycolide)/montmorillonite nanoparticles for oral delivery of anticancer drugs. Biomaterials.

[14] Bromberg L. (2008). Polymeric micelles in oral chemotherapy. J. Control. Release.

[15] Zhang Z., Feng S.S. (2006). Nanoparticles of poly (lactide)/vitamin E TPGS copolymer for cancer chemotherapy: Synthesis, formulation, characterization and in vitro drug release. Biomaterials.

[16] Kalaria D.R., Sharma G., Beniwal V., Ravi Kumar M.N. (2009). Design of biodegradable nanoparticles for oral delivery of doxorubicin: In vivo pharmacokinetics and toxicity studies in rats. Pharm. Res..

[17] Ke W., Zhao Y., Huang R., Jiang C., Pei Y. (2008). Enhanced oral bioavailability of doxorubicin in a dendrimer drug delivery system. J. Pharm. Sci..

[18] Kim J.E., Cho H.J., Kim J.S., Shim C.K., Chung S.J., Oak M.H., Yoon I.S., Kim D.D. (2013). The limited intestinal absorption via paracellular pathway is responsible for the low oral bioavailability of doxorubicin. Xenobiotica.

[19] Natarajan K., Xie Y., Baer M.R., Ross D.D. (2012). Role of breast cancer resistance protein (BCRP/ABCG2) in cancer drug resistance. Biochem. Pharmacol..

[20] Louisa M., Soediro T.M., Suyatna F.D. (2014). In vitro modulation of P-glycoprotein, MRP-1 and BCRP expression by mangiferin in doxorubicin-treated MCF-7 cells. Asian Pac. J. Cancer Prev..

[21] Yin W., Xiang D., Wang T., Zhang Y., Pham C.V., Zhou S., Jiang G., Hou Y., Zhu Y., Han Y. (2021). The inhibition of ABCB1/MDR1 or ABCG2/BCRP enables doxorubicin to eliminate liver cancer stem cells. Sci. Rep..

[22] Shah S.M., Jain A.S., Kaushik R., Nagarsenker M.S., Nerurkar M.J. (2014). Preclinical formulations: Insight, strategies, and practical considerations. AAPS PharmSciTech.

[23] Aungst B.J. (2012). Absorption enhancers: Applications and advances. AAPS J..

[24] Motlekar N.A., Srivenugopal K.S., Wachtel M.S., Youan B.B. (2006). Modulation of gastrointestinal permeability of low-molecular-weight heparin by L-arginine: In-vivo and in-vitro evaluation. J. Pharm. Pharmacol..

[25] Schmidt N., Mishra A., Lai G.H., Wong G.C. (2010). Arginine-rich cell-penetrating peptides. FEBS Lett..

[26] Idrizaj E., Traini C., Vannucchi M.G., Baccari M.C. (2021). Nitric oxide: From gastric motility to gastric dysmotility. Int. J. Mol. Sci..

[27] Kim J.S., Choi W.M., Kim H.I., Chung S.W., Choi J., Lee D., Kim K.M. (2025). Synergistic effects of L-arginine and argininosuccinate synthetase 1 in inducing apoptosis in hepatocellular carcinoma. J. Liver Cancer.

[28] Kummar S., Chen H.X., Wright J., Holbeck S., Millin M.D., Tomaszewski J., Zweibel J., Collins J., Doroshow J.H. (2010). Utilizing targeted cancer therapeutic agents in combination: Novel approaches and urgent requirements. Nat. Rev. Drug Discov..

[29] Bai D., Zhou Y., Jing L., Guo C., Yang Q. (2025). Arginine metabolism in cancer biology and immunotherapy. Immune Netw..

[30] Mocellin S., Bronte V., Nitti D. (2007). Nitric oxide, a double edged sword in cancer biology: Searching for therapeutic opportunities. Med. Res. Rev..

[31] Embaby M.A., El Maghraby G.M., Osman M.A., Sultan A.A. (2023). Investigation of the effect of concurrently administered carvedilol, atorvastatin and bile salts on intestinal absorption of linagliptin. J. Pharm. Pharmacol..

[32] Sultan A.A., El-Gizawy S.A., Osman M.A., El Maghraby G.M. (2018). Niosomes for oral delivery of nateglinide: In situ–in vivo correlation. J. Liposome Res..

[33] Swain L., Das B., Baran N. (2025). Multifaced anticancer potential of doxorubicin: Spotlight on breast cancer. Dis. Res..

[34] Qiu C., Wu Y., Shi Q., Guo Q., Zhang J., Meng Y., Wang C., Xia F., Wang J., Xu C. (2023). Advanced strategies for nucleic acids and small-molecular drugs in combined anticancer therapy. Int. J. Biol. Sci..

[35] Elfadadny A., Ragab R.F., Hamada R., Al Jaouni S.K., Fu J., Mousa S.A., El-Far A.H. (2023). Natural bioactive compounds-doxorubicin combinations targeting topoisomerase II-alpha: Anticancer efficacy and safety. Toxicol. Appl. Pharmacol..

[36] Shu X.L., Liu X.L., Zhong J.X., Liu J. (2014). L-arginine enhances arginine deiminase induced human lymphoma cell growth inhibition through NF-κBp65 and p53 expression in vitro. Eur. Rev. Med. Pharmacol. Sci..

[37] Kabel A.M., Arab H.H., Abd Elmaaboud M.A. (2021). Effect of dapagliflozin and/or L-arginine on solid tumor model in mice: The interaction between nitric oxide, transforming growth factor-beta 1, autophagy, and apoptosis. Fundam. Clin. Pharmacol..

[38] Bani D., Masini E., Bello M.G., Bigazzi M., Sacchi T.B. (1995). Relaxin activates the L-arginine-nitric oxide pathway in human breast cancer cells. Cancer Res..

[39] Herce H.D., Garcia A.E., Cardoso M.C. (2014). Fundamental molecular mechanism for the cellular uptake of guanidinium-rich molecules. J. Am. Chem. Soc..

[40] Kalafatovic D., Giralt E. (2017). Cell-penetrating peptides: Design strategies beyond primary structure and amphipathicity. Molecules.

[41] Stuehr D.J. (2004). Enzymes of the L-arginine to nitric oxide pathway. J. Nutr..

[42] Martin M.J., Jimenez M.D., Motilva V. (2001). New issues about nitric oxide and its effects on the gastrointestinal tract. Curr. Pharm. Des..

[43] Xu D.Z., Lu Q., Deitch E.A. (2002). Nitric oxide directly impairs intestinal barrier function. Shock.

[44] Jahani M., Azadbakht M., Norooznezhad F., Mansouri K. (2017). L-arginine alters the effect of 5-fluorouracil on breast cancer cells in favor of apoptosis. Biomed. Pharmacother..

[45] Scott L., Lamb J., Smith S., Wheatley D.N. (2000). Single amino acid (arginine) deprivation: Rapid and selective death of cultured transformed and malignant cells. Br. J. Cancer.

[46] Yamaki T., Kamiya Y., Ohtake K., Uchida M., Seki T., Ueda H., Kobayashi J., Morimoto Y., Natsume H. (2014). A Mechanism Enhancing Macromolecule Transport Through Paracellular Spaces Induced by Poly-L-Arginine: Poly-L-Arginine Induces the Internalization of Tight Junction Proteins via Clathrin-Mediated Endocytosis. Pharm. Res..

[47] Hirose S., Waku T., Tani M., Masuda H., Endo K., Ashitani S., Aketa I., Kitano H., Nakada S., Wada A. (2023). NRF3 activates mTORC1 arginine-dependently for cancer cell viability. iScience.

[48] Chen C.L., Hsu S.C., Ann D.K., Yen Y., Kung H.J. (2021). Arginine signaling and cancer metabolism. Cancers.

[49] Cai X., Shang L., Li Y., Cao Y., Shi F. (2026). Arginine Transporters in Human Cancers: Emerging Mechanisms and Clinical Implications. Biomolecules.

[50] Ren D., Liu R., Li W., Li L., Lv X., Xia X., Lei X. (2025). Amino acid metabolic reprogramming drives pathogenesis and therapy in hematologic malignancies. Discov. Oncol..

[51] Sultan A.A., El-Gizawy S.A., Osman M.A., El Maghraby G.M. (2016). Colloidal carriers for extended absorption window of furosemide. J. Pharm. Pharmacol..

[52] Sultan A.A., El Nashar N.F., Ashmawy S.M., El Maghraby G.M. (2022). Cubosomes for enhancing intestinal absorption of fexofenadine hydrochloride: In situ and in vivo investigation. Int. J. Nanomed..

[53] Sultan A.A., El-Gizawy S.A., Osman M.A., El Maghraby G.M. (2017). Peceosomes for oral delivery of glibenclamide: In vitro in situ correlation. J. Drug Deliv. Sci. Technol..

[54] Osman M.A., El Maghraby G.M., Hedaya M.A. (2006). Intestinal absorption and presystemic disposition of sildenafil citrate in the rabbit: Evidence for site-dependent absorptive clearance. Biopharm. Drug Dispos..

[55] Poochikian G.K., Cradock J.C., Flora K.P. (1981). Stability of anthracycline antitumor agents in four infusion fluids. Am. J. Hosp. Pharm..

[56] Sadeghi-Aliabadi H., Minaiyan M., Dabestan A. (2010). Cytotoxic evaluation of doxorubicin in combination with simvastatin against human cancer cells. Res. Pharm. Sci..

[57] Cory A.H., Owen T.C., Barltrop J.A., Cory J.G. (1991). Use of an aqueous soluble tetrazolium/formazan assay for cell growth assays in culture. Cancer Commun..

[58] Berridge M.V., Herst P.M., Tan A.S. (2005). Tetrazolium dyes as tools in cell biology: New insights into their cellular reduction. Biotechnol. Annu. Rev..

[59] Rahdar A., Sargazi S., Barani M., Shahraki S., Sabir F., Aboudzadeh M.A. (2021). Lignin-stabilized doxorubicin microemulsions: Synthesis, physical characterization, and in vitro assessments. Polymers.

[60] Chen X., Lv P., Liu J., Xu K. (2009). Apoptosis of human hepatocellular carcinoma cell (HepG2) induced by cardiotoxin III through S-phase arrest. Exp. Toxicol. Pathol..

